# Neurosurgical Management of Skull Base Epidermoid Tumors in Children

**DOI:** 10.7759/cureus.32701

**Published:** 2022-12-19

**Authors:** Marco Antônio Schlindwein Vaz, Joel Lavinsky, Ricardo Santos, Francisco Braga, Paola Santis Isolan, Giuseppe Casella Santis, Gustavo Rassier Isolan

**Affiliations:** 1 Department of Neurosurgery, The Center for Advanced Neurology and Neurosurgery (CEANNE), Porto Alegre, BRA; 2 Department of Neurotology, Clinica Lavinsky (The Center for Neurotology and acoustic neuroma - CNNA), Porto Alegre, BRA; 3 Department of Pediatric Surgery, Rio Grande do Sul Federal University (UFRGS), Porto Alegre, BRA; 4 Department of Pre-medicine, University of North Georgia, Dahlonega, USA; 5 Department of Neurosurgery, Paraná Mackenzie Evangelic University (FEMPAR), Curitiba, BRA

**Keywords:** pediatric surgery, neurosurgery, skull base, pediatrics, epidermoid tumors

## Abstract

Epidermoid tumors (ET) are congenital and benign tumors that develop from de ectoderm during neurogenesis. In the skull base, these lesions can insinuate themselves into several intracranial compartments by filling the subarachnoid space, and possibly affecting some important structures, like the brainstem, cerebellopontine angle, the pituitary in the middle fossa, and others. In the pediatrics skull base tumors, the ET represents 7-9%, being very rare. The surgical perspective of these cases is dependent on the extension of the resection. We presented two cases of total and near-total resection of ETs in the pediatric skull base, with successful outcomes.

## Introduction

Epidermoid tumors (ET) are congenital and benign tumors that develop from de ectoderm during the neurogenesis between the third and fifth weeks of gestation. Its cysts consist in encapsulated forms of epidermoid and connective tissue that grow independently at the expense of its peripheral structures, especially bones [1]. The sac is made of stratified squamous epithelium and contains epithelial debris [2].

These tumors, especially the base skull ones, have many potential cellular origins [1]. Besides that, due its location, these lesions can insinuate themselves into several intracranial compartments by filling the subarachnoid space, and possibly affecting some important structures, like the brainstem, cerebellopontine angle (CPA), the pituitary in the middle fossa, and the chiasmal region near the skull base [1-3].

ET represents a small part of the central nervous system (CNS) tumors, approximately 1%. However, in pediatric skull base tumors, the ET represents 7-9%, being very rare in the skull base. The surgical perspective of these cases is dependent on the extension of the resection [1,2]. Thus, due to the proximity to important structures, total resection is not always possible. Besides that, skull base anatomy in children has some variations that prevent some skull base approaches.

The objective of this article is to report two cases of base skull ETs in the pediatric population and review the literature on these cases.

## Case presentation

Illustrative case 1

**Figure 1:**
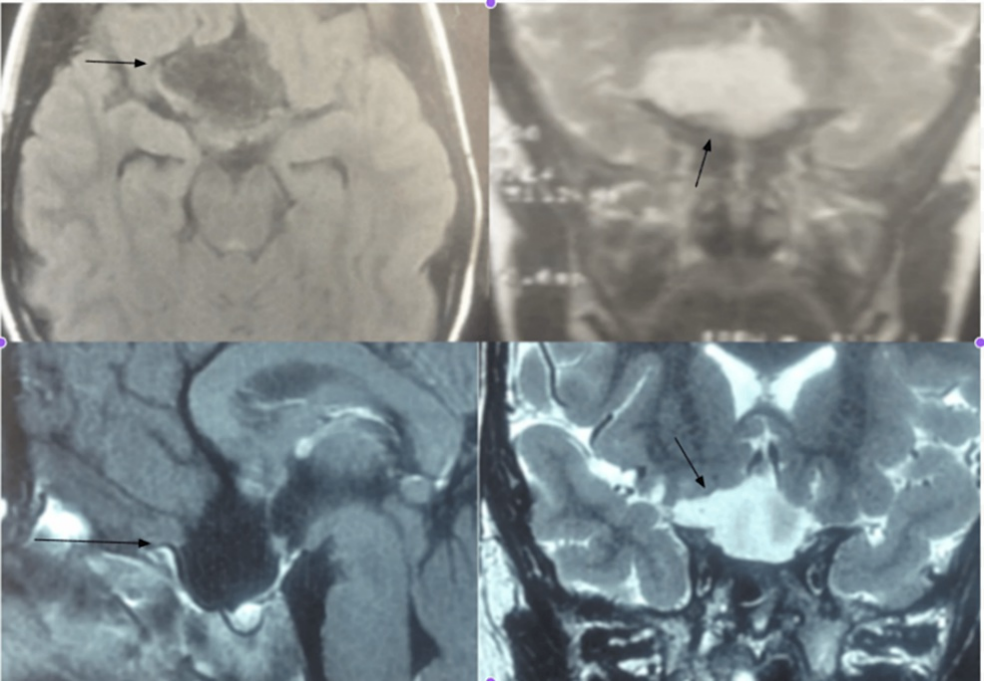
Cranial MRI MRI axial T1-weighted showing hypointense lesions in the parasellar region (top left image). MRI coronal T2-weighted showing hyperintense lesion in the parasellar region (top right image). MRI sagittal T1-weighted with contrast showing the lesion resection with preservation of the pituitary stalk (bottom left image). The resection can also be seen in the MRI coronal T2-weighted (bottom right image).

**Figure 2:**
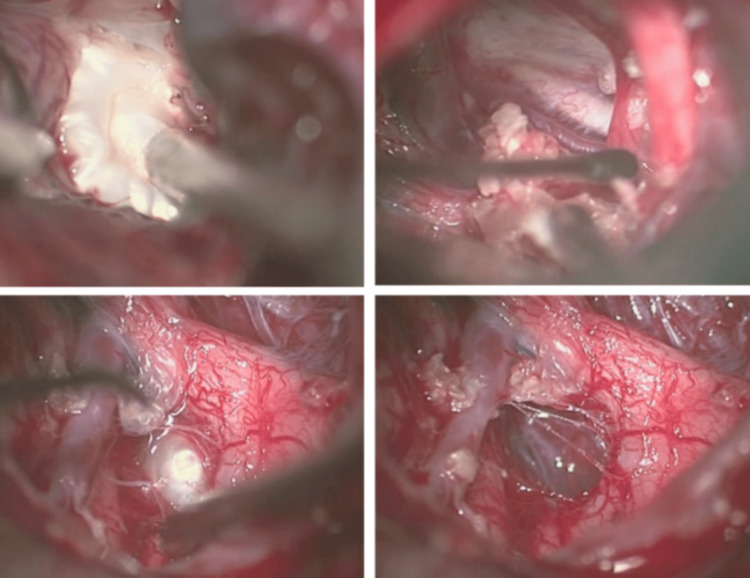
Intraoperative images Image presenting right transsylvian approach showing the tumor enroll with the neurovascular structures and the pituitary stalk (superior left) and after the tumor resection with preservation of the pituitary stalk (superior right). The final part of the tumor located between the optic chiasm at the right and anterior cerebral artery at the left (inferior left), was resected (inferior right).

**Figure 3:**
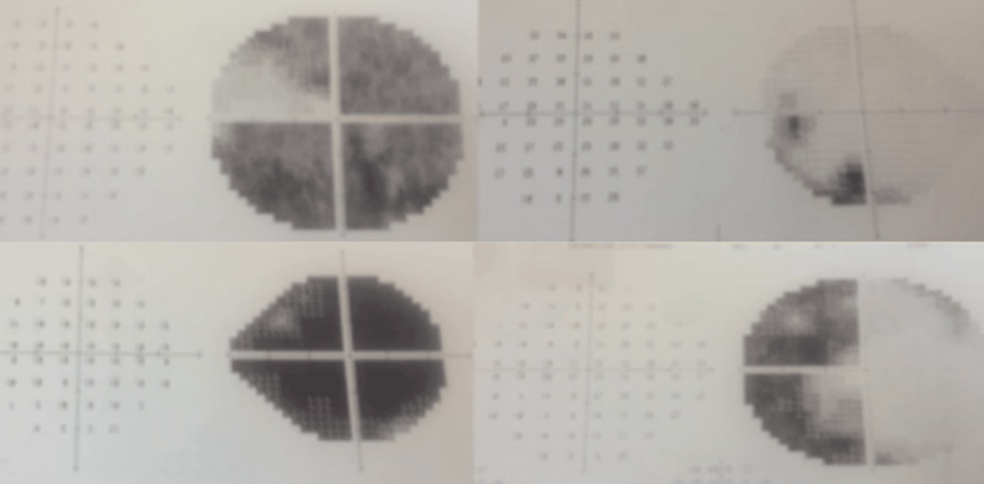
Preoperatory campimetry Campimetry of the right eye (top left image) and the left eye (bottom right image) showing partial vision loss. The right side of the image shows the recovery of the right eye’s vision (top right) and the bottom side image (bottom left) shows the left eye’s recovery after surgery.

A boy, 9 years old, came to our clinic complaining of blurred vision. The cranial MRI showed a tumoral lesion in the suprasellar cistern. The patient was submitted to a right frontotemporal approach, transsylvian with tumor resection (Figures 1-2), improving visual symptoms and campimetry results (Figure 3). The pituitary stalk was encased by the tumor but could be preserved anatomically and functionally through meticulous dissection. A very small part of tumor-related adherent to the anterior choroidal artery was left behind and is being followed up in the last 8 years with a minimum increase in size. There was no postoperative deficit and the endocrinologic function was preserved.

Pathology showed a cystic lesion occupied by keratin lamellae, showing a thin wall consisting of loose connective tissue and lined by keratinized squamous epithelium with keratohyalin granules. The epithelial lining showed positive immunoexpression for cytokeratin (AE1/AE3).

Case 2

A 12-year-old girl came to the emergency department complaining of blurred vision and mild headache for 3 days. Eyes fundoscopy exam showed bilateral papillary edema. Other neurological exams were normal. The hearing was preserved bilaterally. CT showed a hypodense tumoral image in the prepontine and cerebellopontine cisterns plus obstructive hydrocephalus.

**Figure 4:**
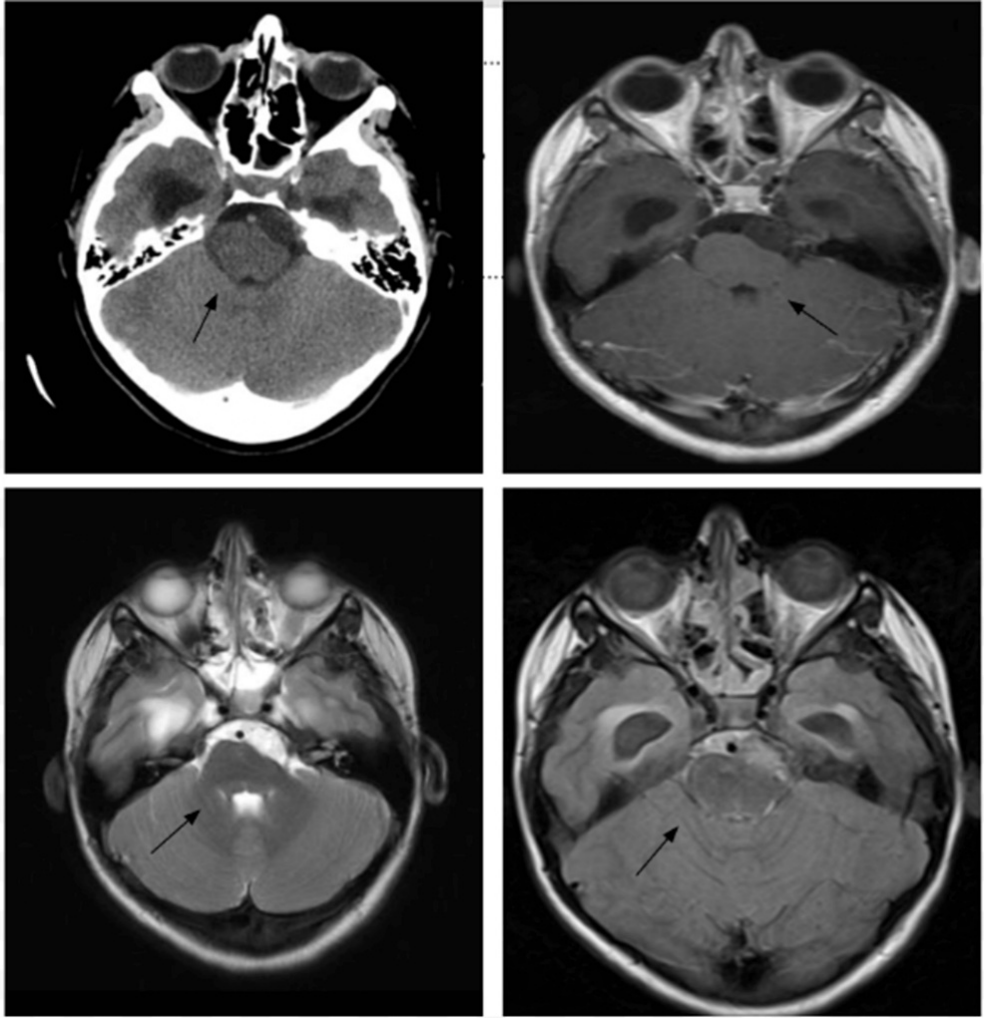
MRI axial T2-weighted images Axial CT scan showing a hypodense mass in the prepontine and cerebellopontine cistern plus hydrocephalus (superior left). This mass was hypointense in T1 (superior right), hyperintense in T2 (inferior left), and hyperintense in FLAIR (inferior right) suggesting an epidermoid tumor. FLAIR: fluid-attenuated inversion recovery

MRI axial T2-weighted image showed a cerebrospinal fluid intensity, extra-axial cystic lesion in the right CPA, and prepontine cistern (Figure 4). Diffusion was restricted, showing hyperintensity on trace DW images.

**Figure 5:**
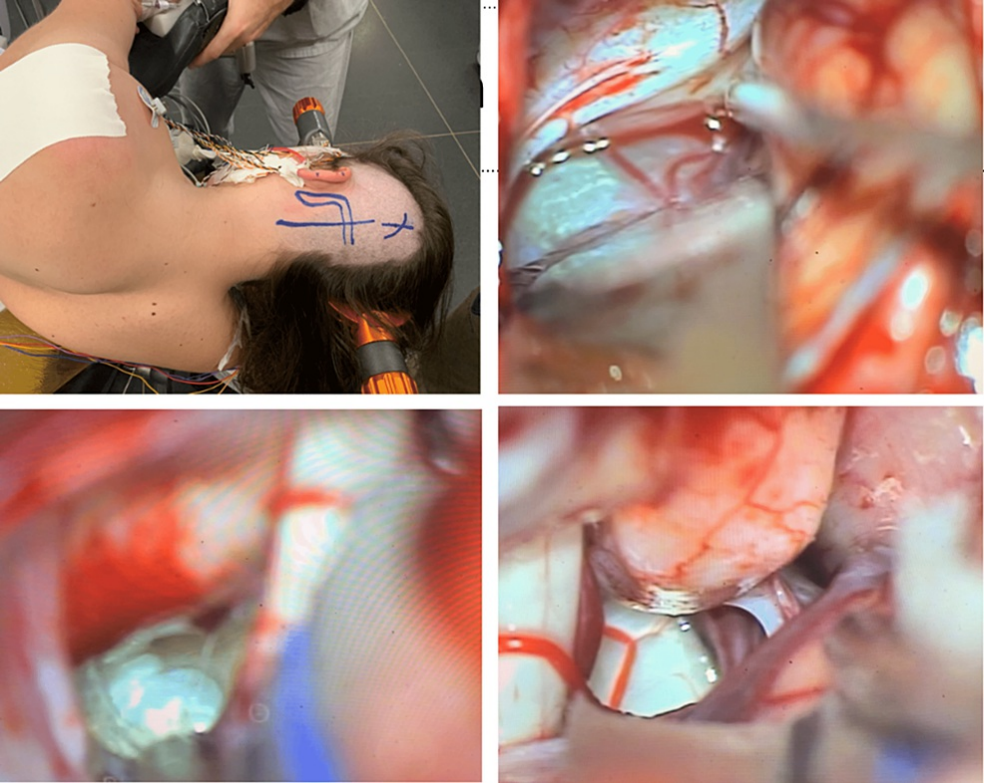
Tumor resection images The patient was positioned in a park bench position (superior left) and a retrosigmoid approach was performed. The arachnoid membrane of the cerebellopontine cistern was thicker than normal (superior right) and was opened to expose the tumor between the lower cranial nerves and the VII-VIII complex (inferior left). The tumor was resected, and an endoscopy was placed to look for tumoral residue. the superior part of the operative field between VII and V CN was not opened (inferior right).

**Figure 6:**
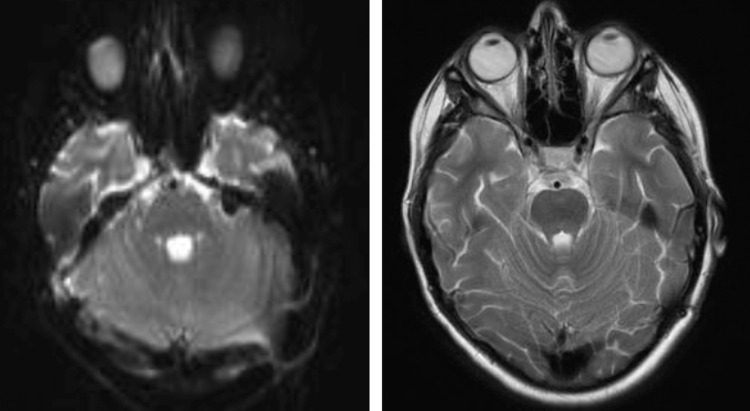
Postoperative showed total tumor resection in FLAIR (left) and MRI T2 (right). FLAIR: fluid-attenuated inversion recovery

The patient was operated on with intraoperative monitoring of the lower cranial nerves, facial nerve, Brainstem Evoked Response Audiometry (BERA), SPSS, and motor-evoked potential. A retrosigmoid approach was performed just after an external drainage catheter was inserted in the right frontal horn of the lateral ventricles and CSF was sent for study. The tumor was resected through the window between the lower cranial nerves and the VII/VIII nerves (Figure 5). There was no postoperative deficit. The CSF study was normal and a ventriculoperitoneal shunt was placed 4 days after this surgery due to no improvement of symptomatic hydrocephalus after tumor resection. The patient was discharged one week after the first surgery. An MRI in the immediate postoperative and 3 months after surgery showed total resection (Figure 6).

## Discussion

ETs originate from ectoderm cells between the third and fifth weeks of gestation, due to the incomplete cleavage of the neural ectoderm. These tumors grow slowly via a physiologic division of epidermoid cells. Thus, symptoms appear when the tumor has already reached a significant size [1].

ET has some specific characteristics in neuroimage. The CT shows a low-density lesion without enhancement. The MRI presents hypointense cists in T1-weighted and hyperintense in T2-weighted [4-10]. This appearance is similar to those found in arachnoid cyst imaging [1,4-5,10].

The most common clinical presentation of base skull ETs in pediatric patients is the involvement of cranial nerves (CN) and cerebellar dysfunction [1]. Some investigators report the involvement of the seventh CN unilaterally and hearing loss [2,6-7]. Headache, mental status alteration, and trigeminal neuralgia are also reported [1-2,6-7].

Meningitis can be present, especially linked to spontaneous rupture of the cyst releasing irritative fluid [1-2]. The rupture of the cyst in the perioperative period is also possible. In our second case, we assumed at first that the patient had meningitis, but the CSF study was normal. In patients with meningitis, the so-called aseptic meningitis, this event can be reduced with the administration of perioperative corticoids and hydrocortisone irrigation in the surgical field [1].

The ideal treatment for skull base ETs is total resection. Nonetheless, the possible proximity and enhancement with import structures and eloquent areas can hinder this gold standard treatment [8]. Some investigators report that only 50-80% of the ET can be resected [9].

Small ET can be resected with the translabyrinthine approach, but in the attempt to audition and facial CN preservation, the retrolabyrinthine approach can be done. Also, mastoidectomy can be performed [2-10]. Some structures that ET can reach are neurovascular structures, CN, perforating arteries, veins, and brainstem [3]. In our second case, since the auditory function was preserved, we contra-indicated the translabyrinthine approach. In the pediatric population, it is frequent that the surgical field achieved in the Trautmann’s triangle to be narrow due to the proximity of the sigmoid sinus and posterior semicircular canal.

Understanding the possible results that a large-size ET in the skull base of pediatric patients, the early detection of this kind of lesion is highly recommended. In small-size tumors, total resection is more likely to be achieved, and the clinical features of the patient are preserved [2-3,10].

## Conclusions

The presented cases show the total and/or near-total resection of a skull base ET in the pediatric population.
